# Utilizing Black Soldier Fly Larvae to Improve Bioconversion and Reduce Pollution: A Sustainable Method for Efficient Treatment of Mixed Wastes of Wet Distiller Grains and Livestock Manure

**DOI:** 10.3390/molecules28155735

**Published:** 2023-07-28

**Authors:** Tao Li, Samiullah Khan, Mao Wei, Haiyin Li, Tingchi Wen, Jianjun Guo, Daochao Jin

**Affiliations:** 1 Institute of Entomology, Guizhou University, Guiyang 550025, China; litaojzx@126.com (T.L.); samiullahakbar4@gmail.com (S.K.); weim111@163.com (M.W.); hyli3@gzu.edu.cn (H.L.); 2 Scientific Observing and Experimental Station of Crop Pest in Guiyang, Ministry of Agriculture and Rural Affairs, Guiyang 550025, China; 3 State Key Laboratory Breeding Base of Green Pesticide and Agricultural Bioengineering, Key Laboratory of Green Pesticide and Agricultural Bioengineering, Ministry of Education, Guizhou University, Guiyang 550025, China; tingchiwen@yahoo.com

**Keywords:** sustainable waste management, black soldier fly, livestock manure, wet distiller grains, fluorescence spectra, humic acid

## Abstract

Widespread environmental contamination caused by huge amounts of wastes generated by human activities has become a critical global concern that requires urgent action. The black soldier fly (BSFL) has gradually been used to treat different wastes due to high efficiency and low cost. However, little information is available regarding the treatment of mixed wastes by BSFLs. The impact of BSFLs on conversion of cow manure (COM) and pig manure (PM) via the incorporation of wet distiller grains (WDG) was assessed. Results demonstrate that the waste reduction rate was increased by 20% by incorporating 45% WDG to COM and PM. The bioconversion rate of BSFLs in COM and PM also increased from 1.20 ± 0.02% and 0.92 ± 0.02% to 10.54 ± 0.06% and 10.05 ± 0.11%, respectively. Total nitrogen content and δ^15^N/^14^N ratios of WDG + COM and WDG + PM were found to be significantly lower than those of COM and PM alone (*p* < 0.01). The organic matter changes during manure degradation were further analyzed by combing ultraviolet–visible spectrum (UV–vis) with excitation–emission matrix (EEM) spectroscopy techniques and fluorescence area integration (FRI) method. The UV–vis spectra results indicate that the addition of WDG to manures resulted in the decreased aromaticity and molecular weight of the waste. EEM spectra demonstrated that the accumulative P_i,n_ values of regions III and V in COM, COM + WDG, PM, and PM + WDG were 58%, 49%, 52% and 63%, respectively. These results not only provide new insights into the potential of mixed wastes for BSFL treatment but also contribute to the basis for the formulation of effective management measurements that reduce and/or reuse these wastes.

## 1. Introduction

Globally, wastes induced by intense animal husbandry have developed to be a serious environmental issue. Increasing wastes derived from the livestock sector, in particular, continues to cause a serious burden for the environment in several developing countries [1]. For example, developing countries generated 2009 million tons of wastes in 2016, representing 64% of the world’s waste; furthermore, the amounts of wastes generated appeared to have higher increased trends in developing countries than those in developed countries [2]. Approximately 587 billion tons of manure were produced by an estimation of the global livestock annually, which contains active organic matter, heavy metals, antibiotic residues, and pathogens, all of which pose a threat to the environment and public health [3,4,5]. It is noteworthy that animal manures contributes up to 40% of the global anthropogenic ammonia (NH_3_) and nitrous oxide (N_2_O) emissions [6,7]. If the hazardous substances in animal manures are not effectively treated, they will severely impact the environment and human health. To improve manure management, 59 million tons of manure was intraregionally transported between farms in Germany in 2015~2016 [8]. However, this approach is uneconomical, requiring lots of manpower and material. Subsequently, several results have demonstrated that composting and/or anaerobic fermentation could effectively treat livestock manures [9,10]. However, the development and application of these technologies have been hindered by various challenges, such as lengthy implementation periods, high costs, low profit, and the production of undesirable gases, particularly NH_3_ and N_2_O [11,12]. Hence, it is urgently needed to develop a more effective and ecologically acceptable method for treating animal manures.

Previous studies have demonstrated that the BSFLs are a sustainable and cost-effective option for the degradation of manures due to their short period of growth and reproduction, enormous biomass, and diverse diet [12,13,14]. BSFLs could decompose the carboxylic, alcohol, and aliphatic components of organic matter, and the carboxylic substances in wastes after black soldier fly treatment increased compared with those before treatment [12,15,16]. Further, comparing the organic matter before and after treatment, the organic matter after treatment obviously decomposed and converted into humic-like substances [17]. In addition, the residues obtained from the bioconversion process of manures could potentially be utilized as fertilizers in agriculture due to their superior humification levels compared with fresh manure, which could have significant benefits for soil health and fertility. Studies have shown that humic substance rates in manures treated with BSFL were significantly higher (42.45–63.74%) than that of raw manures and natural composting manures (38.78–50.31%) [12,18]. Furthermore, BSFLs are also an economic insect, whose larvae can fully mature in 2–3 weeks; compared with the price of fishmeal, each ton of dried BSFL is cheaper, approximately 5800–7800 yuan (data from The China Feed Industry Information Website) [19,20]. Previous studies have reported the low reduction rates of 56% and 12.70% for POM and COM treated by BSFL, respectively [21,22]. Furthermore, subsequent studies also reported the relatively low reduction rates of dewatered sludge and abattoir wastes after being treated by BSFL, approximately 34.93% and 46.30%, respectively [23,24]. However, these studies were limited in scope, focusing mainly on single waste, and they did not account for the potential effects of mixed wastes. Therefore, our study aimed to explore the potential of BSFL bioconversion for mixed livestock manures, such as those containing WDG, which is currently not available.

Furthermore, varied dissolved organic matter (DOM) and nitrogen losses (e.g., the emissions of NH_3_ and N_2_O) caused by denitrification are the most concerned during the biodegradation processes of these manures performed by BSFLs. One is the presence of DOM in the waste, which might serve as a precursor to carcinogenic disinfection induced by byproducts such as trihalomethanes and haloacetic acids [25,26]. The other, namely NH_3_ emissions from animal manures during the biodegradation performed by BSFL, has been recognized as a major environmental issue, which may lead to acid rain and water eutrophication [11,27]. To enhance waste management policies, it is necessary to evaluate the variation of DOM and the rate of N depletion during waste disposal processing. DOM characteristics have been studied using integrated approaches, such as parallel factor analysis (PARAFAC) and fluorescence regional integration (FRI) technique [25,28]. The strongly correlated relationships among the aromatic degree, molecular weight, and hydrophobicity of DOM have been established using the molar absorbance coefficient of UV–vis spectra at 250–280 nm [29,30,31]. Furthermore, significant correlation among SUVA_260_, the degree of humification aromaticity, and hydrophobicity was observed [32]; SUVA_280_ reflected the aromatic compound content in organic matter [33]. In addition, the E_250_/E_365_ ratio was inversely proportional to the degree of humification, and the molecular weight of organic matter was typically correlated with the degree of aromatic condensation and the molecular weight of DOM [30]. Moreover, a previous study further demonstrated that the initial DOM in the residues mainly consisted in the form of biological metabolites, such as soluble microbial products and tryptophan; subsequently, the humic acid (region V) and fulvic acid (region III) became predominant over time, while metabolic intermediates (region IV) were gradually decreased during these periods [34]. Additionally, two studies revealed that protein analogs and soluble microbial byproducts from livestock manures could be metabolized by BSFLs and produce humic substrates [11,35]. However, these results were obtained by integrating many studies with distinct experiment conditions, which could result in incorrect conclusions. Therefore, the transformation mechanism of DOM from animal manures performed by BSFLs during biodegradation warrants further study. Concurrently, protein-derived substances are associated with N content, and the variations of N content during the process of waste disposal by BSFLs are helpful for the understanding of composition difference of DOM. Nitrogen content has been found to be correlated with the presence of harmful gases in animal wastes, as noted in a recent study where BSFL larvae were effective in reducing the nitrogen contents in fresh pig manure [36]. Further, Chadwick et al. (2015) demonstrated that that at least 13% of NH_3_-N from pig manure could be incorporated into BSFL bodies in the form of protein using the stable isotope ^15^N in NH_3_, thus reducing N_2_O emissions [37]. In turn, few studies have compared the variations in N content during disposal different wastes via BSFL [11,35].

To enhance the treatment efficiency for cow and pig manures by BSFL, WDG was added in the two above-mentioned manures. The treatment efficiency of this method was further estimated, and the emissions of nitrogen-related gas were also assessed by TN content and δ^15^N/^14^N ratio. In addition, we solely determined residual DOM from the treated WDG-added livestock manures to evaluate the difference in DOM and the degree of humification of residues using fluorescence technology and FRI analysis. These results help better understand the process of DOM transformation during BSFL treatment and provide a reference for increasing the bioconversion efficiency of livestock manure.

## 2. Results

### 2.1. BSFL Treatment Efficiency for WDG-Added Livestock Manures

In the present study, WDG were mixed into COM and PM to investigate the biodegradation treatment efficiency performed by BSFLs over a twelve-day period. As shown at Table 1, the results show that larval survival rates in the COM + WDG and PM + WDG were 89.08 ± 0.14% and 94.00 ± 0.25%, with growth rate index (GRI) values of 8.53 ± 0.13 mg/day and 7.71 ± 0.14 mg/day, respectively. In contrast, the larval survival rates in solely COM and PM were 65.42 ± 0.29% and 66.58 ± 0.14%, with GRI values of 1.40 ± 0.39 mg/day and 1.16 ± 0.07 mg/day, respectively. After adding WDG, the larval survival rate in the combined COM and PM increased by 23.66% and 27.42%, respectively, and the GRI value increased by 6.09–6.65 times. These results indicate that WDG could significantly increase the survival rates and development of BSFLs in livestock manures. Compared with cow manure treatment, PM + WDG could provide more nutrition, which is conducive to the growth of BSFLs, and hence the BSFL survival rates in the combination of WDG + PM was higher (Table S1). Similarly, after 12 days of the treatments, the reduction rates of the wastes from COM, COM + WDG, PM, and PM + WDG were 43.85 ± 0.74%, 73.41 ± 2.12%, 42.70 ± 2.43% and 67.11 ± 2.52%, with bioconversion rates of 1.20 ± 0.02%, 10.54 ± 0.06%, 0.92 ± 0.02% and 10.05 ± 0.11%, respectively. Overall, these results suggest that WDG could promote the reduction rates and the bioconversion rates of the wastes between these combinations treated by BSFLs.

### 2.2. The Reduction in N-Relevant Harmful Gas Emission with the Addition of WDG in Livestock Manures Treated by BSFL

**Figure 1:**
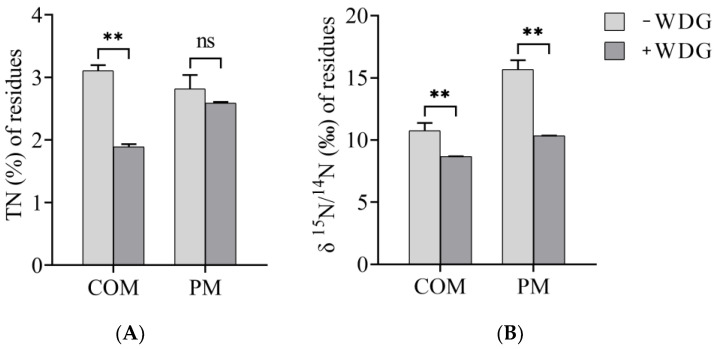
N-relevant harmful gas emission of BSFL bioconversion in residues of livestock manures: TN content (**A**) and δ^15^N/^14^N ratio (**B**) of residues. Each point represents the mean ± standard error (n = 3). ** *p* < 0.01; ^ns^ *p* > 0.05 (determined by one-way ANOVA followed by the LSD test). ANOVA, analysis of variance; −WDG represents manures without WDG; +WDG means feces with WDG.

The variation of TN content and δ^15^N/^14^N ratios in the residues of these treatments was further measured. After the wastes were digested by BSFLs, the TN content of the wastes from COM + WDG (1.89 ± 0.04%) after treatment was substantially lower than that of the solely WDG waste (3.11 ± 0.09%) (Figure S1). By contrast, there was little difference between TN content in the residual PM (2.82 ± 0.22%) and that in PM + WDG (2.59 ± 0.02%) (Figure 1A). Further, the δ^15^N/^14^N ratio in these signal wastes were significantly lower than in these combinations (Figure 1B).

### 2.3. Effect of Adding WDG in Livestock Manures on the Organic Matter

Next, we detected livestock manures with or without WDG using EEM and UV–vis spectra and then carried out FRI technical analysis to further study the effect of adding WDG to feces on organic matter.

#### 2.3.1. EEM Spectra

**Figure 2:**
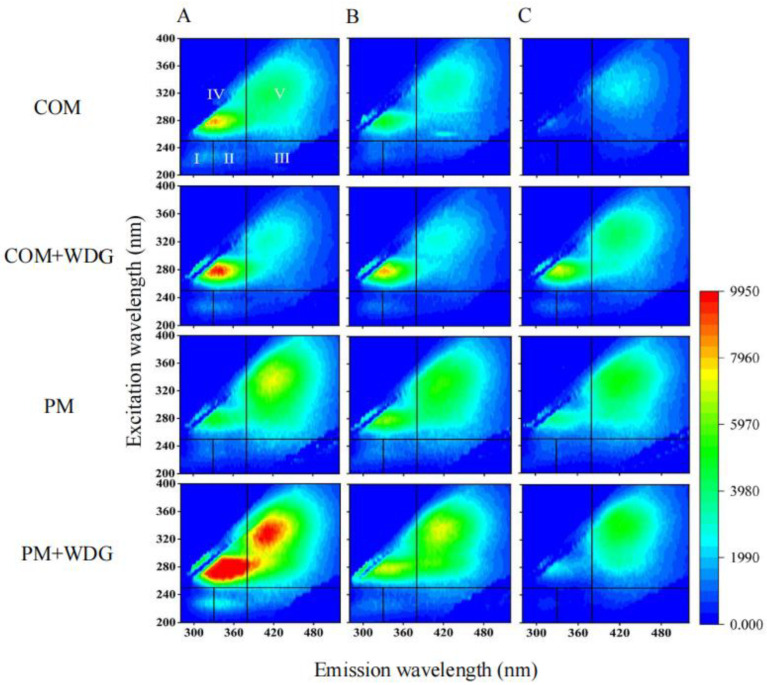
EEM spectra of all samples of DOM in the fresh livestock manures (**A**), control (**B**), and black soldier fly treatment manures (**C**). The excitation/emission wavelength ranges of regions (I, II, III, IV, and V) were 230–250/280–330 nm, 230–250/330–380 nm, 200–250/380–520 nm, 250–440/280–380 nm and 250–440/380–380 nm, respectively. The control group represents natural composting, and the treatment group represents using BSFL degradation waste.

Results from EEM spectra revealed that the fluorescence intensity of regions I and II in mixed samples COM + WDG and PM + WDG of the group C was higher than that of samples of solely COM and PM, indicating that WDG could increase the livestock manures’ aromatic protein content (Figure 2A). After BSFL bioconversion, fluorescence intensity of regions I, II, and IV of group C became weaker than groups A and B, as well as whether or not added WDG had no impact on the overall trend of these three regions (Figure 2). At the same time, the fluorescence intensity of region V of COM + WDG was increased, indicating the generation of humic substances analogues in COM + WDG.

**Figure 3:**
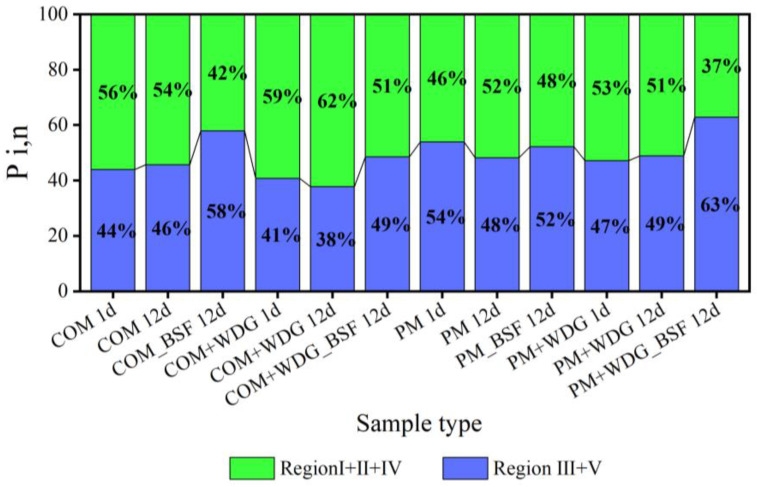
P_i,n_ of the five regions of all samples of DOM from different treatments.

The content of aromatic proteins, fulvic-acid-like substrates, soluble microbial-byproduct-like compounds, and humic-acid-like materials were visualized for each region by calculating their percentages of fluorescence response (P_i,n_) (Figure 3). The accumulative P_i,n_ values of regions I, II, and IV of COM, COM + WDG, and PM + WDG in group C after being treated by BSFLs were markedly lower than those of groups B and A, and the accumulative P_i,n_ values of regions III and V were fundamentally higher than those of groups B and A. The results illustrate that BSFLs accelerated the consumption of protein-like substances and soluble microbial-byproduct-like compounds, as well as promoted the stabilization and humification of organic matter in livestock manures. As for group C, the accumulative P_i,n_ values of regions III and V in COM, COM + WDG, PM, and PM + WDG were 58%, 49%, 52%, and 63%, respectively, suggesting that the level of livestock manures’ humification of COM was decreased by adding WDG.

#### 2.3.2. UV–Vis Spectra

**Figure 4:**
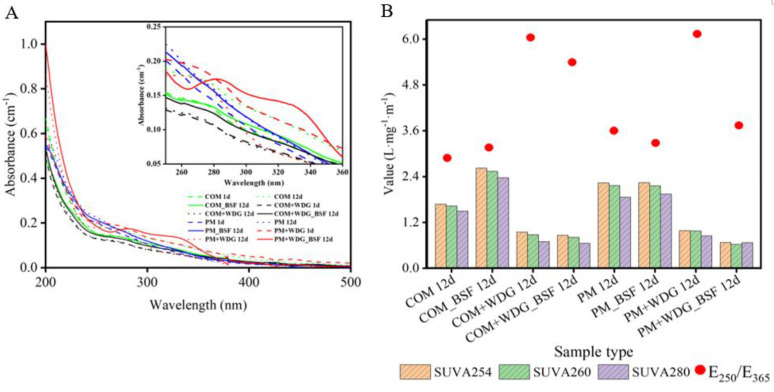
UV−vis spectra (**A**) of DOM in the initial groups, control, and treatment groups and UV spectral coefficient parameters (**B**) of the control and treatment groups.

Next, to further investigate the effect of adding WDG in manures on the varied contents of organic matter, organic matter from these restudies treated by BSFL were analyzed by using UV–vis spectra by molar absorbance coefficients and four representative parameters (SUVA_254_, SUVA_260_, SUVA_280_, and E_250_/E_365_) (Figure 4). After twelve days of being treated by BSFL, the order of the molar absorbance coefficients of DOM from these residues at the range of 250–280 nm was PM + WDG > COM + WDG > COM > PM (Figure 4A), demonstrating that WDG could enhance the decomposition of livestock manures. Subsequently, the SUVA_254_, SUVA_260_, and SUVA_280_ values of the treated groups were found to be significantly higher than the control. However, regardless of whether WDG was added or not, the E_250_/E_365_ ratio of the treated groups was lower than the control, suggesting that the wastes’ aromaticity, molecular weight, hydrophobicity, and humification level when treated by BSFL were higher than natural composting. Above all, WDG did not alter the general trend of organic matter decomposition (Figure 4B). Moreover, the SUVA_254_, SUVA_260_, and SUVA_280_ values in livestock manures with the addition of WDG were lower than those without WDG, but the E_250_/E_365_ ratio was higher in COM + WDG and PM + WDG than in COM and PM, and WDG decreased the aromatic polycondensation, molecular weight, and polar functional groups of DOM in livestock manures.

### 2.4. Phytotoxicity Study and Micronutrient Composition of Residues

To assess the quality and phytotoxicity of the residual manures, edible rape seeds were used to evaluate residues plant toxicity (Table S2). The seed germination index (GI) of all samples was 154.76%, 123.64%, 131.97%, and 164.46%, respectively. Based on the phytotoxicity analysis, we found that all residues proved to be nontoxic and have the potential to be used as soil organic fertilizer. Further, we also tested the micronutrient composition of manure/compost produced by BSFLs, and the results show that the micronutrients included major elements (P, Ca, K, Mg, and Na) and trace elements (Zn, Cu, Fe, Mn, Mo, Ni, Co, and Cr) in residues, and most trace elements were lower than international soil safety standards (except Zn, Fe, and Mn) (Table S3). These residues might be used to improve soil nutrition due their rich nutritional content and low environmental pollution.

## 3. Discussion

### 3.1. Enhancement in BSFL Bioconversion and Livestock Manures’ Reduction Rates

BSFLs have the ability to survive and grow in a variety of substrates of livestock manures [12,34]. However, the reduction and bioconversion rates of wastes from manures were essentially low when treated by BSFL, particularly for COM and PM [12,34]. These findings emphasized that it is urgently needed to optimize the treatment of livestock manures with BSFLs to enhance its efficacy as a sustainable waste management solution. In this study, we investigated the effect of adding 45% WDG into livestock manures on the reduction rates of wastes, and the results demonstrate a significant improvement in the effectiveness of BSFL treatment. The reduction rates of COM and PM without WDG were 43.85 ± 0.74% and 42.70 ± 2.43%, respectively, whereas the addition of WDG increased the reduction rates to 73.41 ± 2.12% and 67.11 ± 2.52% for COM and PM, respectively. Additionally, the BSFL bioconversion rates in COM (1.20 ± 0.02 to 10.54 ± 0.06%) and PM (0.92 ± 0.02 to 10.05 ± 0.11%) were also increased. Compared with cow manure treatment, PM + WDG could provide more nutrition, which was conducive to the growth of BSFL; hence, the addition of WDG in PM could improve BSFL survival rates (Table S1). Similar results were also observed by mixing food wastes into dewatered sludge, with an increased waste reduction rate of 34.93 to 40.75% [3,4,5]. Adding high-nutrient wastes to low-nutrient wastes could increase the combined nutrients and help improve the efficiency of BSFLs in wastes treatment [12]. Fruits and vegetable wastes were added into abattoir waste, which improved the reduction rate from 46.3% to 61.1% [24]. Similarly, adding soybean curd residue into cow manure, the bioconversion rate increased approximately two times, from 6.40% to 11.60% [38]. With BSFL biotreated mixed food waste, the bioconversion rate was the highest, 14.00% in the mixed wastes of coconut endosperm and soybean curd residue and 18.54% in the soybean curd residue and kitchen waste mixture [39,40]. Compared with the single-waste treatment, mixed wastes could provide more nutrition, which is conducive to the growth of BSFLs. Like other living organisms, BSFLs need nutrients to support their growth. Therefore, for higher bioconversion performance, BSFLs need to feed on organic wastes rich in digestible nutritive substances. After treating waste, the larvae could be used to feed poultry, due to BSFLs having polyunsaturated fatty acids and low heavy metal concentrations (heavy metal concentrations were even below limits of international guidelines for animal feed) [13,41]. For example, BSFL could positively increase the levels of the health-claimable omega-3 fatty acid EPA within broiler bodies, and plasma triglyceride concentrations from broiler bodies also increased in a dose-dependent manner with increasing levels of BSFL [41,42]. Additionally, after wastes were treated by BSFL, heavy metals (Cd, Co, Cr, Cu, Fe, Mn, Mo, Ni, Pb, and Zn) were not bioaccumulated in the larvae [13]. According to the study by Shi et al. (2022), these elements had a low chance to bioaccumulate/biomagnify in BSFL bodies due to multiresistance bacterial strains in the gut of BSFLs, which helped the larvae against heavy metal stress [43].

However, the waste reduction rates of solely COM and solely PM were only 12.70% and 56.00% after being treated by BSFL, respectively [21,22]. Hence, we obtained a common conclusion that different mixed wastes could enhance the reduction rate of these wastes. At the same time, in this study, the total Kjeldahl nitrogen of the raw material of WDG (46.11 ± 0.06%) was higher than that of COM (43.17 ± 0.12%) and PM (44.48 ± 0.09%), indicating that WDG contained more protein than the livestock manures. This is consistent with the finding that protein is a necessary nutrient for larval growth and development [44]. Furthermore, the C/N ratio of raw material from WDG (26.44 ± 0.31%) was higher than that of COM (12.91 ± 0.10%) and PM (12.68 ± 0.28%). Previous studies have shown that a higher initial C/N ratio could lead to improved composting stability when mixing chicken manure with different amounts of sawdust [23]. Furthermore, we found that heavy metal levels in WDG were lower than those in COM and PM. Therefore, adding WDG to livestock manures can improve the composting conditions and reduce environmental pollution. The incorporation of WDG into livestock manures resulted in an approximately 20% improvement in waste reduction rates after BSFL treatment. These findings underscored the potential of WDG as an additive to enhance the performance of BSFL treatment in mixed livestock manure.

### 3.2. The Reduction in NH_3_ and N_2_O Emission

While NH_3_ emissions from livestock manures are also one of the main environmental burdens, at the same time, NH_3_ was an indirect source for N_2_O [11,45]. Therefore, to further understand the emission of pollutants (e.g., NH_3_ and N_2_O) during the experiment of treatment by BSFL, it was necessary to emphasize the changes in N contents. In the present study, the TN content and δ^15^N/^14^N ratio of livestock manures before and after the experiment were analyzed. WDG was added into the livestock manure, which significantly decreased TN content and δ^15^N/^14^N ratio in these blended livestock manures, with TN content and δ^15^N/^14^N ratio from COM + WDG and COM being 1.89 ± 0.04% and 3.11 ± 0.09%, 8.68 ± 0.01‰ and 10.76 ± 0.61‰, respectively. These results illustrate that a mixture of different wastes could reduce NH_3_ emission pollution more. These findings also highlighted the potential benefits of cotreatment and codigestion of livestock manures and other waste products. Mixing different waste streams might achieve more effective wastes treatment, resulting in decreased environmental pollution. Earlier studies had explained the reason why NH_3_ emissions could be reduced by using BSFL bioconversion waste: approximately 25% of the N in initial fresh pig manure could be accumulated in larvae by a mass balance law [36,46]. A subsequent study further revealed that 13% of the NH_3_-N derived from manures was incorporated into the larval biomass by using the stable isotope ^15^N during manures’ bioconversion with BSFL [11]. This means that BSFLs could reduce NH_3_ emission by 8.97 kg when 1 ton of pig manure is treated by BSFL. And manure treatment decreased greenhouse gas (CO_2_, CH_4_ and N_2_O) emissions from manures in EU countries by 0–17% in 2010 [47]. In our study, TN contents in the combination of COM + WDG was 1.65 times that of pure manure; we guessed that BSFLs might reduce NH3 emission by 21.39%, which means that BSFLs treating 1 ton of cow manure could reduce NH_3_ emissions by 6.65 kg. However, pig manure kept similar patterns with Parodi et al. (2022). Hence, further exploration is required to quantify harmful gas emissions and better understand the underlying mechanisms of the observed reductions between TN contents and δ^15^N/^14^N ratio and explore the potential benefits and limitations of the combination of different wastes after treatment by BSFL.

### 3.3. Identification of the Composition of Organic Matter

The high humus content of livestock manures played a central role in regulating soil fertility. Hence, two spectra were used to analyze the humification degree of feces after these treatments. The EEM spectra could offer more information for the molecular configuration of organic matter from different sources. EEM spectra with the FRI method were employed to analyze all the samples, and the EEM spectra were divided into five regions using the FRI method [4,48]. Each organic matter has its corresponding region. Further, aromatic proteins, fulvic-like materials, soluble microbial-byproduct-like substances, and humic-like compounds fall in regions I and II, region III, region IV, and region V, respectively [49,50]. EEM data suggested that the fluorescence intensity of regions I, II, and IV of all samples in group C became weaker than that of group A and B after being treated by BSFL, which indicated that the proteinaceous substances and soluble microbial-byproduct-like substrates treated by BSFL could be metabolized to humic compounds, as well as whether or not added WDG had no impact on the overall trends of the decomposition of organic matter. These results are further supported by previous studies [11,51], suggesting that the simple structural organic matter obviously decomposed and converted into the humic-like substances after BSFL treatments. Moreover, the accumulative P_i,n_ values of regions III and V in COM, COM + WDG, PM, and PM + WDG after BSFL treatments were 58%, 49%, 52%, and 63%, respectively, demonstrating that the level of livestock manures’ humification of COM was decreased by adding WDG, and the production of humic matter in PM increased.

UV–vis spectra has been widely utilized to characterize the molecular structure of organic matter [33]. More fractions of DOM characterized by UV–vis were observed than those identified by fluorescence spectroscopy. Results from UV–vis spectra indicate that the aromaticity, molecular weight, humification, and hydrophobicity of the residues were decreased after adding WDG between these treatments. This could be attributed to the comparatively low content of humus in WDG, thus reducing the degree of humification of the feces upon addition [52]. Overall, both spectra results suggest that the addition of WDG increased the protein analogue content of the wastes’ raw material and might decrease the level of humification of livestock manures after BSFL treatments.

### 3.4. Non-Phytotoxicity and Rich Micronutrients of Residues

Based on the phytotoxicity analysis, the GI of all residues in edible rape seeds were more than 120% and higher than the Chinese standard for organic fertilizer (GI ≥ 70%, NY525-2021). Hence, these residues were proved to be nontoxic and have the potential to be used as soil organic fertilizer. Further, to date, studies have shown that the seeds of eight species (white radish, cucumber, fruit radish, edible rape, round radish, hybrid cucumber, cress, and Chinese cabbage) were selected as the best choices to conduct phytotoxicity [53]. For example, Wang et al. (2011) reported that GI measured with Chinese cabbage seeds was about 107.2% for composted pig manure [54]. And the GI was about 120% for composted chicken manure [55]. Compared with their composted manure, our residues had higher GI, revealing that our residues favored the growth and reproduction of the plants.

## 4. Materials and Methods

### 4.1. Raw Materials

Pig manure (PM) and cow manure (COM) were collected from a local company (Guizhou Junong Meat Industry Co., Ltd., Guizhou, China), and WDG were provided from Huaxi district, Guiyang city, Guizhou province, China. BSFLs were obtained from a local breeding base in Bijie city, Guizhou province, China. BSFLs were reared in an artificial climate chamber under controlled conditions (temperature: 28 °C and humidity: 70%) until 10 days old as the initial larvae of the experiment. The micronutrient compositions of manures and WDG are shown in Table S1.

### 4.2. Experimental Design

Two livestock manures were compared in this research with and without WDG to investigate the performance of BSFLs that were reared on various feed substrates: COM, COM + WDG in a ratio of 55:45, PM, and PM + WDG in a ratio of 55:45 (PM + WDG). In order to further understand whether the addition of WDG would affect the difference in organic matter during BSFL treatments, two controlled groups were set up for spectral analysis: one control was raw materials (group A), and the second was manures from natural composting without BSFL (group B). Additionally, the bioconversion of manures of BSFLs was group C. The experimental design is shown in Supplementary Material S1.

All samples were individually placed into plastic containers (28 (length) × 20 (width) × 17 (height) cm), and then 150 g (dry matter) of livestock manures was put into each container separately (Table 1). Approximately 0.2 g of BSFLs (10 days old) was added into each container and mixed with each treatment at a larval density of 1 larva/cm^3^. Moreover, each individual was weighed (approximately 6.80 ± 0.87 mg). Furthermore, the feeding rate was equivalent to 24 mg dry matter/larva/day and then incubated in an indoor manual climatic box (each treatment was replicated three times) at the Institute of Entomology, Guizhou University. Meanwhile, all samples were turned over daily and weighed for insect and residual manures [36]. After 12 days, 25% of the larval pupae in the mixtures were observed, and then the experiment was completed. Pupae and larvae were separated from the mixtures and weighed, before storing them at −80 °C [56].

### 4.3. Survival and Growth of BSFLs on Livestock Manures

At the beginning of the experiment, we weighed and counted the selected initial 10-day-old larvae. After the experiment, the final number and weight of the surviving larvae were recorded. The final weight of the residues were calculated by the difference between the total weight of the rearing container, the weight of the empty container, and the weight of mature larvae [13]. The efficiency of bioconversion performed by BSFL, the survival rate and growth rate index (GRI) of larvae, the substrate reduction rate (SR), and the bioconversion rate (BCR) of total organic wastes were calculated according to Equations (1)–(4) [48,57].

GRI (mg/days) = (L_end_ − L_0_)/t (1)

where t is days of rearing; L_0_ is initial weight of ten-day- old larvae; and L_end_ is total weight of mature larvae.

Survival rate (%) = 100 × nL_end_/nL_0_ (2)

nL_0_ is the number of larvae at the beginning, and nL_end_ is the number of larvae at the end

SR (%) = ((S − R)/S) × 100 (3)

S is the weight of total substrate; R is the weight of total residues

BCR (%) = (L_DM_/S_DM_) × 100 (4)

L_DM_ is the total dry weight of mature larvae; S_DM_ is the dry weight

### 4.4. DOM Extraction and Spectral Analysis

To obtain the content of DOM, samples of groups A, B, and C and Milli-Q water were mixed at a ratio of 1:10 (*w*/*w*, dry weight) [58,59] and then shaken at 25 °C for 24 h, followed by centrifuging at 4390× *g* for 15 min. After centrifugation, the supernatant was filtered using a 0.45 μm filter. The organic carbon (DOC) concentration of the filtrate was analyzed by an automated TOC analyzer (elementar vario TOC select, Alimenta, Germany) with three measurements per sample and an analytical precision < 2% [60]. Finally, the filtrate was diluted to 10 mg/L with Milli-Q water according to the measured DOC concentration, and then the samples were stored at 4 °C. Subsequently, these storage samples were further analyzed by ultraviolet–visible (UV–vis) spectra (by UV–vis spectrophotometer (BlueStar A, Beijing Labtech Instruments Co., Ltd. Beijing, China)), and the excitation–emission matrix (EEM) of fluorescence spectra was measured (by a fluorescence spectrophotometer (F-4500, Japan)) [12].

The UV–vis spectra were detected using a UV–vis spectrophotometer with a quartz cuvette with an optical range length of 1 cm in the range of 200–600 nm, while the samples were put into the quartz cuvette, with Milli-Q water used as a blank. Meanwhile, the absorbance ratios at 254, 260, and 280 nm were calculated according to the methods of Song et al. (2015) and Zhang et al. (2022), noted as SUVA_254_, SUVA_260_, and SUVA_280_, respectively [32,33]. The UV absorbance ratios at 250 and 365 nm were noted as E_250_/E_365_.

A fluorescence spectrophotometer (F-4500) was carried out to measure the excitation–emission matrix (EEM) of the fluorescence spectra. The slit widths of the emission and excitation monochromators were both adjusted to 10 nm; subsequently, the scanning speed was set to 1200 nm/min, and the emission and excitation wavelengths were detected in the range of 280–520 nm and 200–400 nm, respectively. Additionally, the increments of excitation and emission wavelengths were adjusted to 5 nm and 2 nm, respectively [61]. Lastly, the FRI method was used to analyze the EEM spectra, and the EEM spectrum of DOM was divided into five contiguous regions (I, II, III, IV, and V), while the excitation/emission wavelength ranges were 230–250/280–330 nm, 230–250/330–380 nm, 200–250/380–520 nm, 250–440/280–380 nm, and 250–440/380–380 nm, respectively. The volume (Φ_i_) beneath region “i” of the EEM was calculated by the following equation [28,59]:

Φ_i_ = ∫_ex_∫_em_I_λex, λem_ d_λex_d_λem_ (5)

For discrete data, the volume (Φi) was expressed by

Φ_i_ = ∑_ex_∑_em_I_λex, λem_∆λ_ex_∆λ_em_ (6)

in which ∆λ_ex_ is the excitation wavelength interval, and ∆λ_em_ is the emission wavelength interval. I_λex, λem_ was the fluorescence intensity at each excitation–emission wavelength pair.

However, since the divided regions were not equal, the volume integral of each region would have some effect on the interpretation of the whole data because of the different regions, and we thus normalized their area of the regions separately to eliminate this effect. The normalized excitation–emission area volumes (Φ_i,n_, Φ_T,n_) and percent fluorescence response (P_i,n_) were calculated as follows:

Φ_i,n_ = MF_i_Φ_i_ (7)

Φ_T,n_ = ∑ ^5^ Φ_i,n_ (8)

P_i,n_ = Φ_i,n_/Φ_T,n_ × 100% (9)

in which MF_i_ is called multiplication factor.

### 4.5. Analysis of TP, TN, and δ^15^N/^14^N

All samples were freeze-dried at −80 °C for several days. The dried samples were ground and sieved through a 200 mesh. These sieved samples were stored at −20 °C for TP, TN, and δ^15^N/^14^N analysis. The content of TP was analyzed using the combustion method. TP and TN content was analyzed using an organic elemental analyzer (vario MACRO, Elimontal, Germany). The δ^15^N/^14^N ratio of these samples was analyzed using a gas isotope ratio mass spectrometer (MAT253, Thermo Fisher, Waltham, MA, USA).

### 4.6. Phytotoxicity Anlysis

We selected edible rape seeds (seed germination rate greater than 98%, Henan Lvyuan Agricultural Technology Co., Ltd., Zhengzhou, China) to assess residue phytotoxicity. First, 10 g of residue (from wastes after being treated by BSFL) and 100 mL deionized water were shaken at 170 rpm for 30 min and then filtered through 0.45 μm filter paper, and the filtrate was collected. Then, seed germination index was determined [53]. Every treatment had three duplicates.

GI = (A_1_/A_2_) × (B_1_/B_2_) × 100 (10)

A_1_ represents the number of germinated seeds in the extract; A_2_ represents the number of germinated seeds in control; B_1_ represents root length in extract; and B_2_ represents root length in control.

### 4.7. Micronutrient Composition Anlysis

The heavy metal content of raw materials and residues was determined. All samples needed to be dried in a freeze-dryer (FDU-2110, EYELA, Tokyo, Japan) and ground through a 200-mesh screen before analysis. The heavy metal content was measured by the method proposed by the Chinese Ministry of Ecology and Environment [62]. The metal concentrations were detected by inductively coupled plasma mass spectrometry (ICP-MS, Perkin Elmer NexION 300X, Waltham, MA, USA).

## 5. Data Analysis

Statistical calculations and data analysis of all samples were performed using SPSS 22.0 (the significance coefficient was set at *p* < 0.05 or *p* < 0.01). The physical and chemical parameters of all samples were also analyzed using Origin 2021, and EEM spectral data were collected and modified with MATLAB 2020a and FRI analysis.

## 6. Conclusions

The major findings were that adding 45% of WDG into livestock manures treated by BSFL could significantly improve waste reduction rates by approximately 20%. The mixture of WDG and manures treated by BSFL might result in decreasing NH_3_ emissions 1.65 times compared with cow manure alone. Our results suggest that the utilization of mixed wastes could also enhance the bioconversion rate of BSFLs and help reduce levels of harmful matter pollution. These findings contribute to a better understanding of the potential benefits of utilizing mixed wastes by the incorporation of WDG wastes with relatively high nutritional value, in order to minimize environmental pollution by accelerating a reduction in low-nutrient-value wastes as a beneficial strategy for sustainable waste management practices. Most importantly, this study provides an economical and efficient method for the treatment of combined wastes.

## Figures and Tables

**Table 1:** Summary of performance data of BSFLs that are reared on various feed substrates.

Treatment	Sample Rate	Total Weight (Dry Weight)	Survival Rate (%)	Growth Rate Index (mg/Days)	Substrate Reduction Rate (%)	Bioconversion Rate (%)
COM	100%COM	150 g	65.42 ± 0.29 ^a^	0.27 ± 0.03 ^b^	43.85 ± 0.74 ^a^	1.20 ± 0.02 ^b^
COM + WDG	55%COM + 45%WDG		89.08 ± 0.14 ^c^	1.22 ± 0.02 ^c^	73.41 ± 2.12 ^c^	10.54 ± 0.06 ^c^
PM	100%PM		66.58 ± 0.14 ^b^	0.16 ± 0.01 ^a^	42.70 ± 2.43 ^a^	0.92 ± 0.02 ^a^
PM + WDG	55%PM + 45%WDG		94.00 ± 0.25 ^d^	1.11 ± 0.06 ^d^	67.11 ± 2.52 ^b^	10.05 ± 0.11 ^d^

Average and standard deviation (n = 3) are displayed. Different letters indicate significant differences among different treatments (*p* < 0.05): cow manure (COM), pig manure (PM), wet distiller grains (WDG), COM blended with WDG with a ratio of 55:45 (COM + WDG), and PM blended with WDG with a ratio of 55:45 (PM + WDG).

## Data Availability

Not sharing research data due to privacy reasons.

## Supplementary Materials

The following supporting information can be downloaded at: https://www.mdpi.com/article/10.3390/molecules28155735/s1, Figure S1: N-relevant harmful gas emission after manure treated by BSFL; Table S1: Selected characteristics of raw materials in this study; Table S2: The phytotoxicity study of waste residues in rape; Table S3: Micro nutrient composition of manure/compost produced by BSFL.

## Availability of Data and Materials

The datasets analyzed in the current study are available from the corresponding author upon reasonable request.
