# Supplementary material for: Utilizing Black Soldier Fly Larvae to Improve Bioconversion and Reduce Pollution: A Sustainable Method for Efficient Treatment of Mixed Wastes of Wet Distiller Grains and Livestock Manure
Source: Molecules. 2023 Jul 28;28(15):5735. doi: 10.3390/molecules28155735 (PMC10421123; doi:10.3390/molecules28155735)
Supplement: Supplementary file 1 [file molecules-28-05735-s001.zip › molecules-2517464-supplementary.pdf]

Table S1. Selected characteristics of raw materials in this study

Parameters	COM	PM	WDG
pH	6.29±0.11	7.69±0.21	4.26±0.22
Total organic carbon (%)	3.27±0.06	3.35±0.07	1.72±0.09
Total Kjeldahl nitrogen (%)	43.17±0.12	44.48±0.09	46.11±0.06
C/N（%）	12.91±0.10	12.68±0.28	26.44±0.31
TOC (mg/L)	44.10±1.49	58.57±0.52	324.94±6.04
Total phosphorus content (μg/g)	13013±43.58	17487.49±27.63	2394.78±14.66
Al (μg/g)	6876.63±11.50	4324.48±9.79	214.33±6.07
Ca (μg/g)	27198.67±70.04	90227.00±103.55	812.98±10.58
Fe (μg/g)	4811.20±4.03	5822.01±19.27	0
K (μg/g)	26129.00±26.00	27909.67±25.58	1813.13±10.96
Mg (μg/g)	10228.33±13.65	12306.67±17.90	1147.20±15.40
Na (μg/g)	12389.33±558.21	12154.67±19.09	445.56±10.30
Cr (μg/g)	11.75±0.38	16.94±1.12	1.71±0.02
Mn (μg/g)	568.78±11.77	401.23±11.87	10.37±0.28
Co (μg/g)	2.17±0.05	2.38±0.01	0.05±0.01
Ni (μg/g)	4.73±0.16	7.04±0.02	0.75±0.03
Cu (μg/g)	34.25±2.32	188.63±6.03	3.65±0.02
Zn (μg/g)	222.93±10.20	536.04±5.73	34.48±0.12
As (μg/g)	17.22±0.34	18.19±0.19	14.76±0.30
Mo (μg/g)	2.54±0.13	2.93±0.03	1.05±0.05
Cd (μg/g)	0.54±0.02	0.55±0.01	0.24±0.17
Pb (μg/g)	6.26±0.09	6.59±0.06	0.97±0.02
Si (μg/g)	1150.65±45.06	683.31±5.38	119.72±0.74
Sr (μg/g)	171.55±5.52	226.99±4.14	6.06±0.02
Sb (μg/g)	0.57±0.02	0.70±0.01	0.93±0.03
Cs (μg/g)	0.83±0.03	0.97±0.01	0.02±0.00
Ba (μg/g)	138.26±4.20	62.85±2.58	2.84±0.01
Li (μg/g)	4.12±0.05	5.10±0.08	0.12±0.01
Be (μg/g)	0.22±0.03	0.26±0.02	0
V (μg/g)	9.94±0.10	7.65±0.03	0.16±0.01
Ga (μg/g)	2.30±0.07	1.74±0.02	0.05±0.00
Ge (μg/g)	1.22±0.04	0.74±0.02	0.02±0.00
Rb (μg/g)	36.18±2.18	45.14±2.48	1.58±0.02
Sr (μg/g)	125.58±6.20	245.02±4.41	4.11±0.01

Among all statistical tests, the IBM SPSS Statistics 26 (SPSS Inc., USA) software was used with one-way analysis of variance (ANOVA) to consider the BSFL bioconversion wastes as well as the question of excessive heavy metals in raw materials.

Supplementary S1: Experimental design of the study.

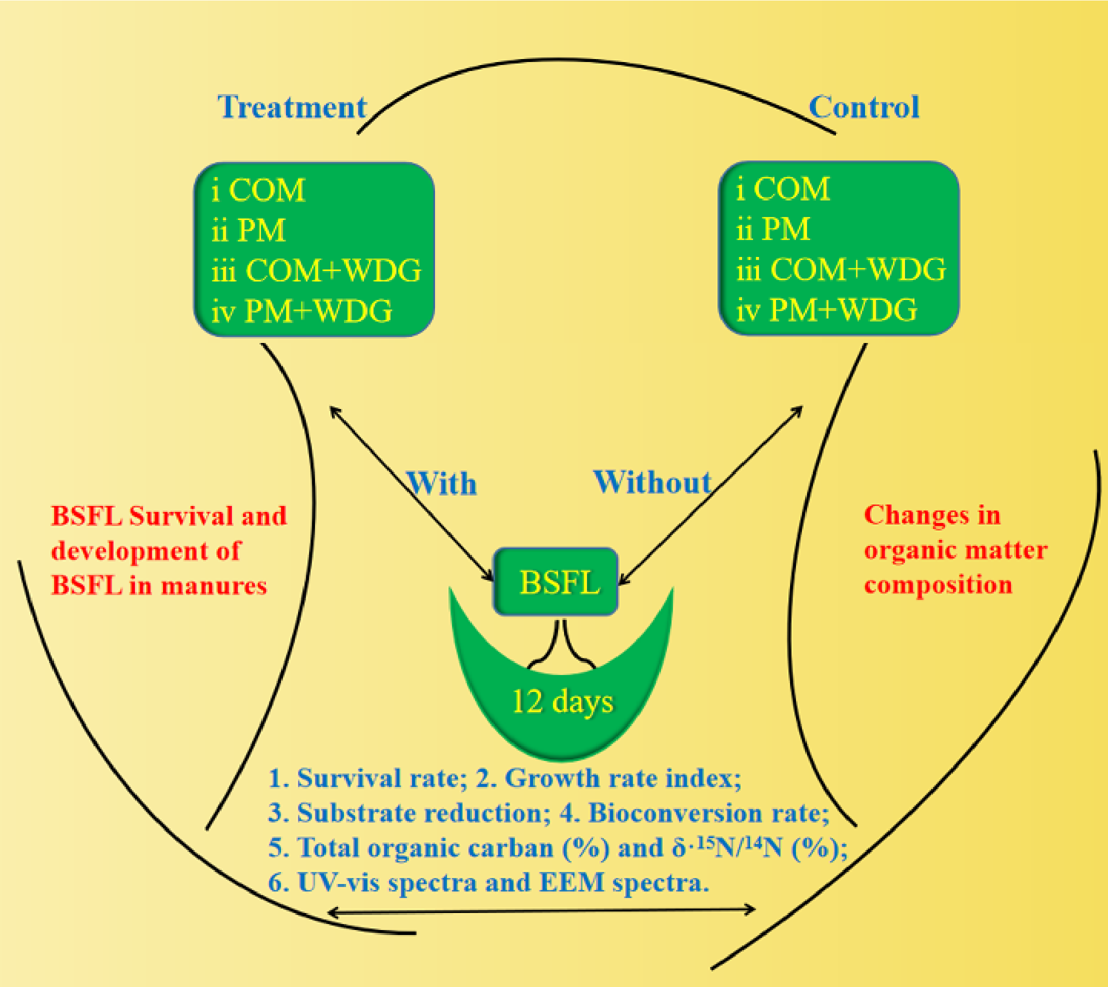

Figure S1: N-relevant harmful gas emission after manure treated by BSFL.

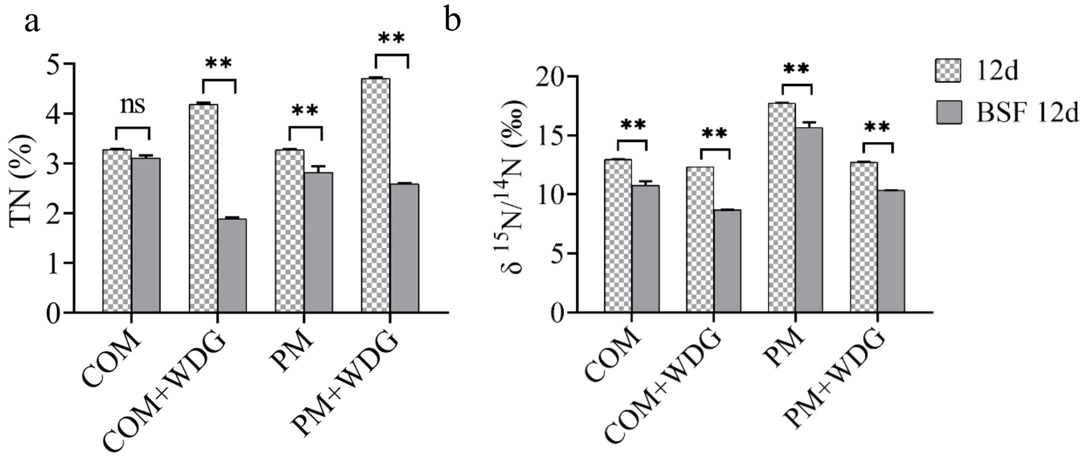

TN content (a) and $\delta^{15}N/^{14}N$ ratio (b) of residual materials in pure and blended manures, **P* < 0.05; ***P*<0.01.

Table S2. The phytotoxicity study of waste residues in rape.

Treatment	Relative germination percentage (%)	GI (%)
COM	100%	154.76±1.45b
COM+WDG	100%	123.64±1.12d
PM	100%	131.97±1.12c
PM+WDG	100%	164.46±1.80a

Average and standard deviation (n = 3) are displayed. Different letters indicate significant differences amongst different treatments ($P < 0.05$).

Table S3. Micro nutrient composition of manure/compost produced by BSFL.

	Major element					Trace element							
Sample	TP	Ca	K	Mg	Na	Zn	Cu	Fe	Mn	Mo	Ni	Co	Cr
COM	14194.21±198.23b	23974.39±61.87c	29253.29±137.47a	12006.00±133.80a	11943.72±105.35a	229.96±5.23d	37.45±1.41b	5250.74±52.63b	638.86±5.74b	3.15±0.09b	20.74±0.84a	2.45±0.18a	46.80±1.02a
COM+WDG	13047.95±115.83d	18417.98±105.98d	25282.33±132.20b	11633.01±112.55a	11302.07±73.44b	339.46±2.92c	30.88±1.47b	4835.69±104.93c	451.24±3.87c	2.63±0.20c	5.24±0.21c	2.13±0.12a	12.07±0.08c
PM	16275.40±110.72a	83025.64±74.94a	22206.66±92.65c	12071.22±106.43a	10537.57±60.88c	419.91±7.13a	173.63±3.91a	6486.80±86.94a	406.97±3.04d	3.65±0.12a	18.43±0.22a	2.42±0.13a	34.08±0.63b
PM+WDG	13546.98±155.09c	52620.26±137.30b	13644.34±135.01d	5476.50±69.32b	3089.24±44.32d	371.61±6.48b	20.09±0.60c	1309.45±72.06d	791.61±1.39a	1.37±0.06d	5.45±0.22c	0.51±0.02b	4.00±0.16d

Average and standard deviation (n = 3) are displayed. Different letters indicate significant differences amongst different treatments ($P < 0.05$).
